# *She knows that she will not come back:* tracing patients and new thresholds of collective surveillance in PMTCT Option B+

**DOI:** 10.1186/s12913-017-2826-7

**Published:** 2018-02-01

**Authors:** Fabian Cataldo, Janet Seeley, Misheck J. Nkhata, Zivai Mupambireyi, Edward Tumwesige, Diana M. Gibb, on behalf of the Lablite team, F. Cataldo, A. K. Chan, L. Chiwaula, M. J. Nkhata, E. Tumwesige, S. Kiwuwa, J. Seeley, G. Abongomera, C. Kityo, H. Namata, J. Hakim, T. Mabugu, M. Muzambi, A. Reid, S. Kaggwa, E. Katabira, I. Mambule, C. F. Gilks, P. Revill, D. Ford, D. M. Gibb, C. Grundy, S. Hoskins, S. Joseph, A. South, M. Thomason, I. Weller

**Affiliations:** 1 Dignitas International, Medical and Research Department, P.O.Box 1071, Zomba, Malawi; 2 London School of Hygiene and Tropical Medicine, 15-17 Tavistock Place, London, WC1H9SH UK; 3 Medical Research Council /Uganda Virus Research Institute, Research Unit on AIDS, P.O.Box 49, Entebbe, Uganda; 4 University of Zimbabwe, P.O.Box MP167, Mount Pleasant, Harare, Zimbabwe; 5 Medical Research Council Clinical Trials Unit at University College London, 90 High Holborn WC1V 6LJ, London, UK

**Keywords:** Community health workers, Tracing, Positionality, Defaulters, Loss to follow up, PMTCT, Option B+

## Abstract

**Background:**

Malawi, Uganda, and Zimbabwe have recently adopted a universal ‘test-and-treat’ approach to the prevention of mother-to-child transmission of HIV (Option B+). Amongst a largely asymptomatic population of women tested for HIV and immediately started on antiretroviral treatment (ART), a relatively high number are not retained in care; they are labelled ‘defaulters’ or ‘lost-to-follow-up’ patients.

**Methods:**

We draw on data collected as part of a study looking at ART decentralization (Lablite) to reflect on the spaces created through the instrumentalization of community health workers (CHWs) for the purpose of bringing women who default from Option B+ back into care. Data were collected through semi-structured interviews with CHWs who are designated to trace Option B+ patients in Uganda, Malawi and Zimbabwe.

**Findings:**

Lost to follow up women give a range of reasons for not coming back to health facilities and often implicitly choose not to be traced by providing a false address at enrolment. New strategies have sought to utilize CHWs’ liminal positionality - situated between the experience of living with HIV, having established local social ties, and being a caretaker - in order to track ‘defaulters’. CHWs are often deployed without adequate guidance or training to protect confidentiality and respect patients’ choice.

**Conclusions:**

CHWs provide essential linkages between health services and patients; they embody the role of ‘extension workers’, a bridge between a novel health policy and ‘non-compliant patients’. Option B+ offers a powerful narrative of the construction of a unilateral ‘moral economy’, which requires the full compliance of patients newly initiated on treatment.

## Background

Since the 1970s, the role of community health workers (CHWs) has increasingly been recognised and formalised through their contribution to public health policies and interventions [1, 2]. A renewed focus on CHWs’ involvement in public health interventions has examined some of the important shifts in the roles of these workers as they take on increasingly specialised tasks in order to support overburdened health care staff, notably in low income settings [1, 3]. More recently, a growing number of social scientists have called for a greater emphasis on the evolving positionality of CHWs, and to further explore the complex dynamics of relationships between CHWs, patients, health care workers, and health systems, as actors embedded in social ties and local moral economies [4–7]. Colvin and Swartz [8] have recently argued that CHWs have been historically framed either as ‘extension agents’ or ‘agents of change’, and that it is crucial to move beyond the ideals of CHWs as either ‘tools’ to solve problems, or ‘magical objects invested with a capacity to solve technical or political dilemmas’ [8].

In this paper, we focus on the experience of CHWs contributing to recent efforts aiming at preventing mother to child transmission of HIV (PMTCT) in low-income countries, namely ‘Option B+’, [9] a health policy under which pregnant and breastfeeding women are routinely tested for HIV and immediately started on antiretroviral treatment (ART) for life after a positive diagnosis, regardless of an individual’s CD4 count or WHO clinical stage [10–12]. Several studies have explored the initial impact of the implementation of this new PMTCT strategy, [13–20] however little attention has been paid to the experience of CHWs who play a central part in supporting patients as well as the larger the health system as part of these interventions.

At the onset of the implementation of Option B+, in 2011 in Malawi, and 2013 in Zimbabwe and Uganda, [9, 14] several public health experts voiced concerns in relation to the challenges of retaining a largely asymptomatic population in care on the long term [21, 22]; A condition of the success of Option B+ is dependent on a high proportion of patients being retained in care. More importantly, perhaps, is the concern around the development of resistance to treatment if newly initiated ART patients do not adhere to life-long treatment. In its first year of implementation in Malawi, the programme had been successful at rapidly expanding access to PMTCT services, with a seven-fold increase in the number of women starting ART for PMTCT, [23, 24] impacting current efforts at expanding ART care, with most patients newly initiated at decentralized health facilities starting ART because of Option B+, as opposed to starting ART for their own health (i.e. because of low CD4 counts or WHO clinical stage) [25].

The first couple of years of implementation of Option B+ in sub-Saharan African countries have demonstrated that women initiating ART during pregnancy have high rates of dropout [16] and retention of women on ART remains a challenge. Initially in Malawi, a large proportion of women (17%) appeared to be ‘lost-to-follow-up’ (LTFU) six months after ART initiation, with most losses occurring in the first three months of therapy [13]. Many women who accepted their first month of ART never came back, raising additional questions on whether or not they actually initiated treatment at all. Early assessments into the reasons associated with stopping ART amongst women who initiated treatment during Option B+ pointed at difficulties linked to travelling to health facilities and lack of transport money, not understanding initial treatment guidance, and being too weak, sick or experiencing treatment side effects [15].

Patients labelled as ‘LTFU’ have been portrayed as ‘threats’ to Option B+. As a result, a rapidly increasing body of research is looking at novel strategies to retain patients in care [26], and locate those who have been labelled as ‘lost’ to encourage them to return into care. ‘Tracking’ HIV patients who have been labelled as ‘defaulters’ is not a new activity however, and a variety of lay, community, home-based and health care workers have previously been involved in finding patients, counselling them and encouraging them to come back into care to resume ART [27–29]. Within Option B+, however, in the light of the potential risks to the programme embodied by ‘defaulters’, the task of tracing patients has become a crucial component of the test-and-treat approach, and CHWs have been called upon to find patients who have missed scheduled visits to the health facilities.

We present data that were collected as part of a sub-study conducted within a large three-country implementation study entitled *Lablite*, which looked at the decentralization of ART care in Malawi, Uganda and Zimbabwe from 2010 onwards, during the time that Option B+ started to be rolled out in these three countries.

## Methods

The aim of this study was to explore the range of tracing strategies deployed in three countries that initiated Option B+, as well as the consequences of ‘tracing’ on social relations between CHWs and patients. Our intention is to describe and discuss the evolving ‘positionality’ of CHWs who are involved in a critical component of new PMTCT programmes: the ‘tracers’, as they are commonly referred to, who are tasked to track ‘non-compliant’ patients with the aim of bringing them back into care. The term ‘positionality’ is used to describe how individuals are defined by their location within shifting networks of relationships [30] and their position (for instance in terms of power, hierarchy, solidarity, control) [31]. In this paper, ‘positionality’ is conceived in relation to the ways CHWs’ roles and identities are formed and altered through changing social networks and relationships involving a range of local actors including, but not limited to, patients and formal health care workers.

The study was designed around qualitative data collection taking place simultaneously in the three countries from December 2014 to June 2015. It was conducted in a sub-set of five health facilities in Malawi (Phalombe District), six in Uganda (Kalungu and Agago districts) and four in Zimbabwe (Zvimba district). We collected data through semi-structured interviews with CHWs who are designated to trace Option B+ patients in each of the Lablite sites in Uganda, Malawi and Zimbabwe.

Study participants were identified through a purposive sampling approach at each study site until the sample of respondents was achieved. In each site, the research team attempted to interview three to six individuals who were tasked with tracing HIV patients under the Option B+ programme. Research participants included a range of CHWs locally designated as Village Health Workers (Zimbabwe), Community Health Volunteers (Uganda), Linkage Facilitators (Uganda), Health Surveillance Assistants (Malawi), and Expert Patients (Malawi). These CHWs were purposively identified to take part in the study because they routinely traced patients as part of PMTCT care activities in the selected health facilities. In total, 60 individuals (29 males, 31 females) were interviewed as part of this study: Uganda *n* = 19 (10 males, 9 females), Malawi *n* = 26 (19 males, 7 females), Zimbabwe *n* = 15 (2 males, 13 females).

In each country, a team of experienced social scientists interviewed research participants. Interviews were conducted at a private location within health facilities or in a private location chosen by research participants. Each interview lasted between 40 and 90 min. The same semi-structured interview guides were used in the three countries. Interview guides focused on tracing strategies, reasons for LFTU, gender dynamics, perceptions of CHWs and ‘tracers’, and perceptions of CHWs’ tasks. In Uganda and Zimbabwe, direct notes, verbatim quotes and observations were compiled in English by the interviewers; due to logistical limitations it was not possible to record interviews. In Malawi, data were recorded on digital audio recorder in the local language (Chichewa), and later fully transcribed and translated into English. Data were fully anonymised and pseudonyms were used to protect the identity of research participants.

Data analysis was conducted primarily through a deductive approach [32, 33]. A thematic framework was formulated to explore issues related to tracing strategies, local definitions of a ‘defaulter’, reasons for patient loss to follow up, relationships with patients and health care workers, perception of tracers and tracers’ perception of their own job, and strategies to reduce loss to follow up. The coding structure was applied to the data using Nvivo10. A narrative account of the findings was developed from the themes and quotes extracted from the coded data.

In the following sections, we employ the term ‘community health worker’ as an umbrella category comprising lay health workers who are members of the communities where they work, who are supported by local organisations or by the national health system where they live, and who have received a shorter training than professional health workers [2].

## Results and discussion

In the following sections, we present a summary of the results and we discuss some of the data from interviews with CHWs in the three countries. We begin with the description of ‘defaulters’, and follow with a discussion of the role and positionality of CHWs in the light of this novel public health programme.

### Who are the ‘defaulters’

The formal definition of a ‘defaulter’ varies by country. The epidemiological categorisation of a defaulter designates an individual who fails to complete a course of treatment, and often carries a negative connotation of blame towards the patient [34]. The term is used by health care workers and CHWs to designate an individual who has been registered or enrolled in a programme and who has not come back for subsequent visits under the same initiative. Under the Option B+ programme, and in the health facilities where we conducted our interviews, patients were designated as ‘defaulters’ after a period varying from one to three months (depending on the country) from the date a patient missed a scheduled appointment to collect ART.

In the study sites, ‘defaulters’ are identified by health care workers who maintain large handwritten registries to document each patient’s visit and the date at which they are asked to come back to pick up treatment or for additional routine tests. CHWs work in collaboration with other health care workers (nurses, ART clerks, clinical officers) in order to identify ‘defaulters’ from the registers and patient cards, which contain basic information about patients (such as the name, and sometimes the address or village, phone number, ART card number, patient register number).

According to recent studies conducted in Malawi [13], and as expressed by the CHWs who we interviewed, defaulters under Option B+ are often younger women who only started treatment a few weeks earlier. One of the main reasons for women to discontinue treatment is linked to dynamics between women who just tested HIV-positive and their male partners. Most women enrolled in Option B+ were routinely tested for HIV at their first antenatal visit. While they were seeking antenatal care, they found themselves offered HIV testing for the first time, and offered to start treatment immediately. Consequently, in the three countries, CHWs provided examples pointing to the difficulties for many women whose partner has not been tested for HIV or, if tested positive, had not started HIV treatment:

It is culturally difficult for women to disclose such difficult matters in as much as it is difficult for a woman to negotiate or initiate condom use in the marriage. So they try to keep it a secret and end up not coming to the clinic to take their medications. (Zimbabwe, Zvimba, BKT003).

Some women don’t come back to pick their treatment because they fear that their husbands will ask them why they swallow drugs every day. Some of these women don’t go to the clinic with their husbands so when they are tested positive they fear to tell them, and when they are started on treatment they again fear to tell them that they are on drugs because their husbands will think they are the ones who went outside and got HIV. (Uganda, Agago, LIK005).

Men are not actively involved in this programme of going to the hospital with their wives during the first antenatal visit. If men were to get tested for HIV and involved, they could be more understanding. Experience has shown that when women receive treatment, they do not take it in the presence of the husband. They take it secretly because by then the husband has not yet accepted her status. Women do not disclose their status at times to their husbands and that is part of the problem. (Malawi, Phalombe, NKH003).

Additional reasons were cited as contributing to defaulting from treatment; In Malawi and Uganda, seven of the CHWs interviewed said that complaints about the behaviour of some of the healthcare workers had contributed to patients leaving treatment; “One client openly said she stopped taking treatment because some health workers insulted her in front of other patients” (Uganda, Bukukula, BUK003).

Giving birth to HIV negative children was also cited by one CHW as another cause for loss to follow up: “After giving birth to HIV free children they [mothers] don’t come back for treatment. Their only aim was to have a child without HIV, that is all” (Uganda, Kalungu, PAI004). Another reason for stopping treatment was linked to religious beliefs and practices. One of the Malawian CHWs commented: “when these women [enrolled in Option B+] put their faith in prayers, they at the same time find it to contradicting to be taking the antiretrovirals for HIV treatment” (Malawi, Phalombe, CTK002). Other CHWs (11 in Malawi and one in Zimbabwe) said that some church representatives and religious leaders advised patients against taking HIV treatment: “There are some churches which according to their doctrines don’t allow their members to go to the hospital when they are sick, instead they just want to pray for them. They say ‘I can’t access ART treatment, I rely on prayers, I depend on Jesus’.” (Malawi, Phalombe, SUH001).

In addition, in the three countries, stigma remained an important concern for patients. They feared that accessing treatment for HIV may jeopardize their reputation and relationships with others living in the same neighbourhood: “People are ashamed of being seen by their neighbours at the clinic […] if you are seen in the queue, then you are HIV-positive” (Zimbabwe, Zimba, NYA004).

A range of other reasons was given in relation to discontinuing treatment and not coming back to the health facility. In Uganda and Zimbabwe, agricultural work during the planting season was mentioned by six of the CHWs. Other reasons included exchanging treatment between patients, self-referrals to other health centres, the lack of understanding of the need to take ART when asymptomatic, side effects, alternative herbal remedies, suspicion in relation to the validity of test results, and the lack of privacy during visits to the health facility. Distance between the patient’s home and the health facility, and the associated costs for transport, were also perceived as an important deterrent to women’s continued access to HIV medication in the three countries.

Hence, to understand the complexity of the work of those involved in tracing patients, it is crucial to recognise and engage with the heterogeneous reasons that led an individual to be labelled as a ‘defaulter’ by the system; These vary from personal experience of being stigmatized, inter-personal and family dynamics, to the cost of transport and the fear of returning to the health facilities. In the next sections, we explore the strategies developed by CHWs to address some of these issues in the context of their effort to bring patients back to care.

### Plural identities

Most of the CHWs who trace patients as part of Option B+ are not new CHWs, nor do they exclusively spend their working time tracing patients. Instead, it is important to note that the term ‘CHW’ is used in this context in relation to a multitude of cadres of lay and formal health workers, as mentioned above. The distinction between lay and formal health workers is often blurred as each cadre participates to various degrees in day to day activities at the health centres where they are based, and the distribution of tasks can greatly vary from one site to another. In addition, each category of CHWs has been set up alongside the advent of specific interventions and health policies, and receives support from various local and global institutions, such as government (Ministries of Health), non-governmental organisations (especially supporting expert patients initiatives), and global partnerships set up to tackle HIV/AIDS. At each study site, lay and formal health workers constituted a heteroclite workforce, organised and defined through varying power dynamics and hierarchical relations between the different cadres of healthcare workers working together. Lay health workers, as described in other settings, [3] have become integrated to the work of formal health workers, especially in the context of the HIV response. The CHWs who we interviewed also reflected these plural identities and did not represent a homogeneous group; they were remunerated, supervised, and supported differently by a myriad of organisations, undertook different training curricula, and had a distinct history of involvement in HIV care and treatment in the three countries.

Many of the CHWs felt personally invested in their work and expressed that they cared deeply for the women and babies who they traced: “in short I can say I am directing her [the patient’s] life” (Malawi, Phalombe, SUH001). They took pride in contributing to "saving lives", as they put it: “One of the most rewarding aspects of my work has been to save the lives of patients with HIV who had lost hope and are now up and strong again” (Uganda, Agago, KAL002). “The biggest rewarding aspect of my work is that the HIV positive patients trust and confided in me for most of their issues related to HIV” (Uganda, Agago, KAL001).

One of the Ugandan CHWs expressed pride in her contribution to getting a patient back into the care system:

Sometimes when doing follow up you find some HIV patients bedridden, discriminated and neglected by their own families so when I help such a patient to get her life back and to have the support from her family I feel so proud of what I have done. I am also happy that I act as a link between the hospital and the community in the HIV services. (Uganda, Agago, KAL008).

Others also expressed a renewed sense of belonging and responsibility in their own community through tracing activities: “I am now very much known in the community, I did not study a lot but at least I am valuable in the community” (Uganda, Kalungu, BUK003).

In Malawi, many CHWs referred to themselves as ‘walking counsellors’. Because of their proximity and access to patients’ private spaces, CHWs said that they hold a privileged position in comparison to health care workers who are based at the health facility:

The patients see us as their doctors; anything or any problem they have, the person can tell us, they are open to us […] they can be free to share with us […] rather than for them to go to the hospital and be in a queue [where] they feel discriminated. When they are explaining to us they are free because they know that this is the person who can help us even when they come back home they say when I see my counselor walking in the village I will be able to explain to her the problems I am facing. (Malawi, Phalombe, MPS003).

In Uganda, many were officially designated as ‘linkage facilitators’: “We link the community to the hospital so each one of their linkage facilitator here is located in the parish or community […] this means that the mothers you follow up in most cases know you and you know them too” (Uganda, Agago, KAL001).

### Peripheral relationships

Because of their prior or current involvements under a variety of other initiatives, many of the CHWs involved in this study were already part of local social networks, and utilised these links in order to trace patients. In the three countries, the reaction from people who were traced was reported by CHWs as positive overall: “Most of the women agreed to come back on HIV treatment and assure us to come back to the health facility on the agreed dates” (Malawi, Phalombe, CTK001).

As already noted above by the CHWs in Malawi, they are often perceived as medical experts by their own communities, with whom they have been working for some time. Some patients and local inhabitants call them ‘doctors’, in reference to their affiliation with health facilities: “they see us as people who give them accurate information compared to that a doctor can give” (Malawi, Phalombe, MPS002). Sometimes, CHWs also received gifts from the women who they visited as a token of appreciation for their visit.

Some CHWs in Malawi and Uganda reported witnessing a change in the way they were perceived locally, which they attributed to the visible features of tracing activities: “At first when we were starting, people were taking us as useless people but after seeing our work they saw that we are important people” (Malawi, Phalombe, NKH003).

On the other hand, CHWs from the three countries reported some tense encounters when they reached the patient who they were looking for. In Uganda and Zimbabwe, some of the mothers who have recently tested positive for HIV reacted with anger at being traced back to their homes: “some women responded wildly when asked to come back to the clinic. They shouted at me and told me to mind about my life, not theirs” (Uganda, Kalungu, KIR008). Younger women, especially, were harder to convince to return for treatment, according to some of the Malawian and Ugandan CHWs:

The most difficult group to deal with are the young mothers. Most of these young mothers and girls don’t want to be associated with the HIV positive people including the health workers and us the linkage facilitators who deal with HIV positive people. I have witnessed some who changed their living places so as to elude me when I am following them up. (Uganda, Agago, KAL002).

Across the study settings, CHWs noted that women who refuse to come back for treatment are often afraid of being stigmatized:

When you ask them [women] to come back to the [health] facility, they willingly tell you ‘thanks so much for coming and for caring about my life, I am going to come to the clinic and continue my treatment’. But actually very few come even after promising that they are coming. (Uganda, Kalungu, BUK004).

Some CHWs are openly living with HIV and referred to themselves as ‘expert patients’. With some patients, CHWs perceived that living with HIV helped them to empathise with the patients’ personal difficulties of adhering to treatment. Other patients were concerned with the risk of stigma that the CHWs' visits represented if these CHWs openly lived with HIV themselves. In Uganda, one of the CHWs who was openly known as living with HIV in his own village expressed: “Some patients hate me as I try to follow them up when they miss appointments, they look at me as a messenger of stigma” (Uganda, Agago, KAL003).

In summary, CHWs reported mixed reactions from patients in relation to being traced back home. Some women were grateful for the opportunity to discuss their difficulties in taking HIV treatment. Many other patients, however, felt uncomfortable and reluctant towards the repeated visits from CHWs at their homes: “she [the patient] looks at you [the CHW] as an enemy, as someone who has got enough time to waste visiting her home or someone who has more than enough money to waste buying [mobile phone] airtime to call her” (Uganda, Kalungu, BUK003).

Through their involvement in tracing activities under Option B+, we argue that CHWs have purposively instrumentalised their liminal positionality, which is situated between the experience of living with HIV (in the case of ‘expert patients’), belonging to local social networks and sometimes representing existing networks, and being perceived as a caretaker or medical specialist. In the following section, we explore some of the strategies developed by CHWs to ‘instrumentalise’ their liminal positionality in the context of tracing patients.

### Refining tracing strategies

In the light of the complex dilemma that they individually face, CHWs have developed a range of individual strategies to get patients back into care. In the three countries, tracing strategies involved visiting patients at home. To do this, CHWs need to first discuss what information is available with the health facility staff. Patients often provide a wrong address when they are being registered, after being tested for HIV or when asked to start HIV treatment. Equipped with often minimal and erroneous information about a patient’s identity and whereabouts, CHWs embark on a daily quest to find Option B+ ‘defaulters’. In the three countries, bicycles were the main means of transport for CHWs, with others having no other option than walking long distances in order to find patients.

In some cases, when a phone number was available and the patient lived far away, some CHWs attempted to call patients, but “we use our own personal money so that we manage to contact the person in question” (Malawi, Phalombe, SUH003). At times, the patient was successfully located through phone calls to friends and family.

In Malawi, if a CHW was not familiar with the area, one of the first steps was often to go through the head of a specific village, and seek permission from the chief to visit a woman at home. CHWs observed that the village chief seemed to be in a position to “give a lot of reasons for their [women] failure to come back to the health facility” (Malawi, Phalombe, CTK001). CHWs also talk to other village members, neighbours, and family members about a specific individual who has defaulted from treatment in order to find them. In Uganda, CHWs reported also talking to patients’ friends at the health facility in order to locate them. “We tend to ask around for her friends […] we ask them to tell us why their ‘colleague’ does not come for treatment” (Uganda, Kalungu, KIR008).

Given the nature of these strategies to trace patients, some CHWs recognized the need to put in place procedures which protect the confidentiality of patients’ serostatus. Most of them said that they did not make specific mention of HIV to others (village head, friends, family members) when tracing patients. One CHW from Malawi, however, expressed that confidentiality was not as important as it once was:

There is no secret nowadays. There was confidentiality in the past but not now as far as these drugs are concerned. […] The people who are HIV positive say that those who are not HIV positive are not in fashion. [By the time they come to the health facility], people who are on ART treatment have already known one another (Malawi, Phalombe, NKH004).

CHWs can spend up to three weeks chasing up a specific patient, attempting to contact them several times: “We go again in the evening so that maybe we find them […] if it fails, we can go for her early that Sunday” (Malawi, Phalombe, CTK006). Many also mentioned that they either sent or left a message with the patient’s family: “We may leave the message home if they are children or her husband we get to leave the message: ‘people from their health facility came here. They say they would like to see you on this date. Try not to go anywhere until they find you'.” (Malawi, Phalombe, CTK006).

One of the Ugandan CHWs reported carrying antiretroviral drugs to dispense them without patients having to travel back to the health facility:

Always we carry some drugs with us when visiting them because we are certain that when a mother misses [a visit] to pick her drugs, they are definitely already out of the ARVs so we can give them. […] we make a follow up on all the patients who promised to return to the health facility for drugs during home visits. When one fails to follow the appointment, we follow them up again and again until they return for treatment (Uganda, Agago, PAI010).

When the CHWs find a patient who has defaulted, they report carrying out one-to-one counselling which aims at convincing the woman to come back into care and to start treatment again. The advice provided often went beyond adherence to treatment, as they provided advice on relationships and talked to male partners about testing for HIV: “we go to their home and chat with the husband until their husband decides to come in the open and test” (Malawi, Phalombe, CTK005).

### Beyond tracing

Some CHWs reported that they threatened non-complying patients, and continued to check on them several months after their initial contact. In Zimbabwe, one of the CHWs said: “I ask [to see] their medication to make sure that they have gone to the clinic to collect their medication and I also [do] a physical count of the drugs. We inform the women that ‘if you don’t go to the clinic we will report you to the [health facility] head’ and people will eventually go to the clinic to avoid being fined […]” (Zimbabwe, Zvimba, NYA001).

Home visits are not only necessary in some settings where there are no other possible options to trace patients, they were also favoured by many of the CHWs as an opportunity to check on the patients. In Zimbabwe, one of the CHWs confided: “face-to-face is good as you can assess the situation for yourself unlike over the phone […] they might be lying but if you visit them in their homes you will be able to tell if they are really sick. Over the phone people can come up with a lot of excuses but if you visit them then they may be honest with you” (Zimbabwe, Zvimba, NYA005).

When patients come back for treatment at the health facility, some of the CHWs continue to actively check on them in collaboration with nurses “to check on the number of ARVs given so that we can ably monitor our patients for them not to default again” (Malawi, Phalombe, CTK001). They continue to follow up these patients through phone calls, and if this fails, additional home visits. “We don’t just stop there after tracing her [patient], we do continue following up on this person till she starts coming to the health facility to pick up ART and to monitor how she is taking up the antiretrovirals.” (Malawi, Phalombe, CTK002).

In Malawi, several CHWs had set up a system to follow-up on patients on the long term. One of them explained:

We agree on a specific date on which they [patients] can come [to the health facility]. When that date comes, we go and visit them again to find out if they have gone to the clinic. […] I check their health passports and some would actually show me the medicine and give me the details of their visit to the clinics. […] It does not end there; if a woman […] is pregnant or has just given birth, we continue giving them home visits for some extended time up until the baby is two years old (Malawi, Phalombe, MPS001).

Another CHW from Malawi added:

When these women come to the hospital to resume treatment […] we request that they bring out bottles of ARVs as evidence that they indeed resumed treatment. We also check their health passports for evidence of the same. […] if the woman was being stigmatized, we ask her what the current state of affairs is with regards to that issue of stigma (Malawi, Phalombe, MPS002).

One of the CHWs in Malawi detailed her approach to confronting patients who have defaulted from care:

Once we get the patient details, we go out to trace the woman. Once found, we have to entice her until she agrees to come back. We don’t just approach her and ask why she has stopped taking her medication. If we did that, she wouldn’t accept. But we talk to her gently. We ask for her health passport, and usually they will give it to us. As you go through the health passport, you are looking for information as to whether she really got tested and was found HIV positive and whether she is indeed on treatment. Now the women cannot refuse that the health passport isn’t hers and at the same time, you will have found the evidence you had been looking for (Malawi, Phalombe, CTK003).

Some CHWs also double checked in the health registers to ensure that a woman has come back for treatment: “we check […] the registers to see if she really came back or maybe she just accepted [to visit the health facility] with the aim of making us leave her house but, from deep down, she knows that she will not come back” (Malawi, Phalombe, SUH001). A strong collaboration with health facility staff enabled CHWs to efficiently follow up each patient individually, sometimes for reasons beyond the retention in Option B+: “We are continuously in touch with the community coordinator at the health facility. […] We handle adherence and social problems in the lives of HIV/AIDS patients. We counsel them when facing difficulties in their marriages or in their villages” (Uganda, Agago, KAL002).

Thus, CHWs involved in tracing patients under Option B+ have developed a range of strategies for tracing. Our observations in the study sites were that CHWs often rely on their own financial resources (phone airtime, bicycles, transport costs, time) to find patients, and lacked supervision, guidance and training specific to the respect of patient confidentiality as part of Option B+.

The strategies developed by CHWs, who are themselves pressurised to successfully bring back patients into care, may tend to reinforce a type of support which relies on recurrent and intimate ‘surveillance’ and monitoring of patients, at times pressurising patients into providing medical information about themselves, whilst remaining suspicious and threatening to report and fine those who do not comply with the request to come back into care. Even when patients complied to come back into care, CHWs ensured that they actively followed up prior defaulters for up to two years. Some of these strategies can seem problematic in relation to the respect of the privacy and confidentiality of patients who may have implicitly opted out from Option B+ by providing erroneous contact details at registration.

### Limitations

Several limitations must be noted in relation to our approach. The data collected were cross-sectional and limit our ability to describe changes in the positionality of CHWs over time and in relation to the evolution of the implementation of Option B+. In addition, the data presented in this paper are limited to a few sites in each country and comprise interviews with diverse cadres of CHWs (village health workers, expert patients, community health workers, health surveillance assistants). As noted in the paper, each of these cadres of CHWs have a different history and function, and we recognise the need to explore in more depth how these different functions and identities affect or influence the actual tasks and perception around tracing activities. Furthermore, despite having selected our study sites with the Lablite team with the intention of including sites which are representative of the current decentralization of ART in the three selected countries, we acknowledge that the views and opinions presented in this paper are not representative of all CHWs in these sites and we have tried as much as possible to indicate to what extent each of the views of CHWs were shared amongst the individuals who participated in the study.

## Conclusion

In this paper, our intention was to explore some of the recent shifts in the positionality of CHWs that have accompanied the implementation of Option B+ and its patient tracing activities. Our approach illustrates how low retention into care and non-compliance to ART are envisaged as threats, not only to the future success of the implementation of this large public health policy, they also jeopardize the treatment outcomes of population groups if new resistance strains to current antiretrovirals were to develop.

Our findings point at similar shifts in the three countries; CHWs who are tasked to trace ‘defaulters’ as part of Option B+ have developed strategies to utilise their liminal positions and identities. On the one hand, they provide essential linkages between health services and patients as they embody the role of ‘extension workers’. Our study attests to the shifts in the role of CHWs described by Kalofonos and Maes as well as others, where CHWs take on the functions of community management, surveillance and reporting of desirable as well as unacceptable behaviours [6, 35]. Here, the role of CHWs as ‘extension workers’ has become conflated with the creation of uncomfortable spaces through the responsibility of bringing back ‘non-compliant’ patients into care.

Yet, the same CHWs also represent a symbolic and operational thread, bridging a novel and quasi-experimental health policy with its main threat, embodied through the non-compliant patients, the ‘defaulters’. In this instance, as Moyer [36] observed in a different context, monitoring and adherence become collective rather than individualized practices [36], contributing, as she put it, to what Nguyen [37] described as an assemblage of pharmaceuticals, services, and surveillance tools [37].

Given the underlying complexities and individual reasons that lead one to ‘default’ from treatment, we question the over-reliance on CHWs, as well as the lack of support they receive, to address what is conceptualised as a ‘threat’ to Option B+. Against the backdrop of a successful and wide-reaching health policy, the act of ‘tracing’ and checking on patients attests to the materialisation of ‘moral contracts’, that is “a set of tacit and explicit expectations underlying relationships between actors involved in the global care continuum” [7]. As CHWs become “front-line care providers at the ‘interface’ between large-scale policy aspirations and local programme implementation, they also become invested with a liability to comply with model targets and operate within more complex, technical systems of care” [7].

A thorough review of the processes involved in test-and-treat processes, including the lack of time and space for patients to make decisions about their own care is urgently needed, as we argue in a separate paper [38]. Option B+ offers a powerful narrative of the construction of a unilateral ‘moral economy’, which requires the compliance of patients who are newly initiated on treatment. In this context, decision-making and therapeutic choice is often negated from patients themselves and CHWs have become liable to make these decisions on behalf of the patients who they are trying to get back into care.

## Abbreviations

ART: Antiretroviral Therapy
ARVs: Antiretroviral Drugs
CHW: Community Health Worker
FGDs: Focus Group Discussions
HIV: Human Immunodeficiency Virus
HSAs: Health Surveillance Assistants
LTFU: Lost to Follow Up
PLHIV: People Living with HIV
PMTCT: Prevention of Mother-to-Child Transmission of HIV
